# Association of plasma biomarkers of Alzheimer’s pathology and neurodegeneration with gait performance in older adults

**DOI:** 10.1038/s43856-024-00713-6

**Published:** 2025-01-16

**Authors:** Farwa Ali, Jeremy A. Syrjanen, Dan J. Figdore, Walter K. Kremers, Michelle M. Mielke, Clifford R. Jack, David S. Knopman, Prashanthi Vemuri, Jonathan Graff-Radford, B. Gwen Windham, Leland R. Barnard, Ronald C. Petersen, Alicia Algeciras-Schimnich

**Affiliations:** 1 Department of Neurology, Mayo Clinic, Rochester, MN USA; 2 Department of Quantitative Health Sciences, Mayo Clinic, Rochester, MN USA; 3 Department of Laboratory Medicine and Pathology, Mayo Clinic, Rochester, MN USA; 4 Department of Epidemiology and Prevention, Wake Forest University School of Medicine, Winston-Salem, NC USA; 5 Department of Radiology, Mayo Clinic, Rochester, MN USA; 6 Department of Medicine, The MIND Center, University of Mississippi Medical Center, Jackson, MS USA

**Keywords:** Medical research, Predictive markers, Predictive markers

## Abstract

**Background:**

Declining gait performance is seen in aging individuals, due to neural and systemic factors. Plasma biomarkers provide an accessible way to assess evolving brain changes; non-specific neurodegeneration (NfL, GFAP) or evolving Alzheimer’s disease (Aβ 42/40 ratio, P-Tau181).

**Methods:**

In a population-based cohort of older adults, we evaluate the hypothesis that plasma biomarkers of neurodegeneration and Alzheimer’s Disease pathology are associated with worse gait performance. A sample of 2641 Mayo Clinic Study of Aging participants with measurements of plasma biomarkers and gait parameters was analyzed in this cross-sectional study. Linear regression models using plasma biomarkers as predictors of gait parameters and adjusted for age, sex, BMI, Charlson Comorbidity Index, and cognitive diagnosis were evaluated.

**Results:**

In this study multiple statistically significant relationships are observed for GFAP, NfL, and P-Tau181 with gait parameters. Each standard deviation increase in GFAP, NfL, and P-Tau181 is associated with a reduction in velocity of 2.100 (95% CI: −3.004, −1.196; *p* = 5.4 × 10^−6^), 4.400 (−5.292, -3.507; *p* = 9.5 × 10^−22^), and 2.617 (−3.414, −1.819; *p* = 1.5 × 10^−10^) cm/sec, respectively. Overall, NfL has the strongest associations with poor gait performance. Models with age interactions show that the strength of associations between the plasma biomarkers and the gait parameters became stronger with increasing age. There are no specific gait parameters that associate with individual plasma biomarkers.

**Conclusion:**

Plasma biomarkers of neurodegeneration and Alzheimer’s Disease pathology are not only markers of cognitive decline but also indicate motor decline in the aging population.

## Abstract (web summary)

Ali, Syrjanen et al. examine the relationship between plasma biomarkers of Alzheimer’s disease pathology and neurodegeneration and their impact on gait performance in older adults. Elevated biomarkers correlate with diminished gait functionality, suggesting motor function decline is indicated alongside cognitive impairment in the aging population.

## Plain language summary

Decline in gait and balance occurs as an individual gets older. These changes can increase risk for falls, death and disability. However, the brain changes that cause a declining gait and balance in aging are incompletely understood. Here, we evaluated gait performance in over 2000 individuals as well as blood biomarkers that indicate evolving Alzheimer’s disease or neurodegeneration. We found that gait changes in the aging individual are associated with neurodegeneration and accumulation of Alzheimer’s disease pathology independent of their memory performance. These findings offer evidence for the role of blood biomarkers in investigating gait decline. Future research will build upon these findings to expand our understanding of brain mechanisms that contribute to gait and balance abnormalities in aging individuals.

## Introduction

Gait and balance function incorporates several neural locomotor and cognitive brain systems that can be affected by neurodegeneration^1,2^. Gait decline in older individuals predicts impending morbidity from falls, nursing home placement, dementia and mortality^3–6^. Motoric decline is an important issue as rates of fatal falls in individuals over 65 years approximately doubled from 1999 to 2020 going from an age-adjusted mortality rate of 29.4 deaths due to falls per 100,000 people over age 65 per year in 1999 to 69.4 in 2020^7^. The impact of disability from gait changes is predicted to increase with the growth of the aging population^8^. Motoric decline in an aging individual is multifactorial, but degeneration in distinct brain regions both in the cortical gray and connecting white matter is thought to play a role^9,10^.

Plasma biomarkers can measure by-products of non-specific neurodegeneration (NfL or GFAP) or specific neuropathologies like Alzheimer’s disease (Aβ 42/40 ratio, P-Tau181). Herein, we assess the relationship of plasma biomarkers with gait and balance function in older adults. Plasma biomarkers are less-invasive, more cost-effective, and more convenient to collect relative to biomarkers measured in cerebrospinal fluid (CSF) or through neuroimaging, such as magnetic resonance imaging (MRI) or positron emission tomography (PET) scans. Plasma biomarkers for amyloid and tau pathology, closely reflect Alzheimer’s disease-related neuropathological burden on PET imaging^11–14^ and in CSF^15^, and are associated with clinical cognitive decline^16–18^. Markers of axonal and astrocytic degeneration, such as neurofilament light chain (NfL) and glial fibrillary acidic protein (GFAP), respectively, albeit nonspecific, are markers of neuronal injury and reflect neurodegeneration^19,20^. Whether these plasma biomarkers are associated with motor decline in older adults is incompletely understood. When considering associations between these plasma biomarkers and gait parameters it is important to consider systemic factors that may be important confounders, affecting both gait performance and biomarkers levels. Renal dysfunction, for example, can affect the peripheral clearance of plasma biomarkers of Alzheimer’s Disease (AD) and neurodegeneration, hence affecting their levels, therefore medical comorbidities such as chronic kidney disease (CKD) should be taken into consideration when exploring these relationships^21–25^.

Few prior studies have explored the relationship between plasma biomarker levels and gait parameters. One study found no evidence of cross-sectional or longitudinal association between plasma amyloid-beta (Aβ) 42/40 ratio or NfL with gait speed in a sample of 507 individuals from the Multidomain Alzheimer’s Preventive Trial^26^. Another study, which utilized a large sample from the Framingham Heart Study Offspring Cohort, found that faster walking speed was associated with lower plasma total tau levels^27^. Gait decline in older adults is multi-factorial. The question remains whether plasma biomarkers of neurodegeneration and AD pathology reflect motor decline in older adults after controlling for age, sex, cognitive state, and medical comorbidities. While causation cannot be determined in a cross-sectional study, we sought to explore possible neural contributors to motor decline by assessing the relationship between plasma biomarkers of neurodegeneration and AD neuropathology in older adults. Gait is a complex multifaceted process, often quantified with different gait domains such as pace, rhythm, variability, and postural control^28^. Therefore, we hypothesized that analyzing diverse gait parameters, that can capture distinct properties of gait, in conjunction with a range of plasma biomarkers, may shed light on how early neurodegeneration (measured by NfL and GFAP) and AD neuropathology (measured by Aβ 42/40 ratio and P-Tau181) impacts motor performance. This premise is supported by previous work showing the differential impact of neurodegeneration on gait domains^29–31^. Our goal was to assess whether observed relationships provide insights into the neuropathological processes driving gait impairment in a cohort of older adults based on plasma biomarker profiles. In this study, we find that gait parameters associated with plasma biomarkers of AD pathology and neurodegeneration independently of cognitive status. These relationships get stronger with age and may differ between men and women.

## Methods

In the present study, Aβ 42/40 ratio, GFAP, NfL, and phosphorylated tau 181 (P-Tau181) levels were measured in plasma specimens from a sample of 2641 Mayo Clinic Study of Aging (MCSA) participants. Gait parameters were measured using a pressure-sensitive walkway (GAITrite^TM^ or Zeno^TM^ ProtoKinetics) at or near (i.e., within a few days) the study visit corresponding to the plasma draw. We used linear regression models to evaluate relationships. Our models were controlled for important confounders such as age, sex, BMI, medical comorbidity burden (Charlson comorbidity index), and cognitive diagnosis (cognitively unimpaired-CU or cognitively impaired-CI). We also assessed how age, sex, CKD, and cognitive diagnosis each modifies observed relationships using interaction-based models. We hypothesized that higher levels of plasma biomarkers of neurodegeneration and AD (i.e., higher NfL or P-Tau181), would be associated with worse gait parameters (i.e., slower gait speed). Our exploratory hypothesis was to assess whether different gait parameters associated with distinct plasma biomarkers of neurodegeneration versus AD.

### Mayo Clinic Study of Aging (MCSA)

The MCSA is a population-based study employing an age and sex-stratified random sampling scheme selected from those living in Olmsted County, Minnesota. Those consenting to study participation come into the clinic for a visit consisting of a complete neurological and neuropsychiatric exam administered by trained neurologists, neuropsychiatrists, and study coordinators. Additionally, a blood draw is performed as well as CSF collection through lumbar puncture, MRI, and PET imaging for those agreeing to participate in those aspects of the study. A clinical diagnosis of CU^32–35^, MCI^36,37^, or dementia^38^ is assigned to each participant at each visit based on published diagnostic criteria and further discussed at a consensus conference amongst experts in aging, dementia, and staff involved in examining the participant. The study protocol was approved by the Mayo Clinic and Olmsted Medical Center institutional review boards (IRB #14-004401). Complete study details can be found elsewhere^39^. Written informed consent was obtained from all participants or their legally assigned representative.

### Plasma

Participants fasted overnight before plasms specimen collection. EDTA-plasma samples were collected and centrifuged. Aliquots of 500 μL each of plasma were stored at −80 °C in polypropylene tubes until testing. Simoa® Neurology 4-Plex E Advantage kit (N4PE, item #103670) was used to measure Aβ 1-40, Aβ 1-42, GFAP, and NfL. Simoa® pTau-181 Advantage V2 kit (item #103714P-Tau181) was used to measure pTau-181. Test was conducted per manufacturer’s instructions and the plasma sample was analyzed using a Quanterix HD-X analyzer (Quanterix, Lexington, MA, USA). The exact testing protocol included thawing and mixing of the plasma samples followed by centrifugation for 5 minutes at 4000 *g*. For each sample, 1:4 dilution was performed using the onboard dilution protocol and tested in singlet. A seven-point calibration curve and sample concentrations were determined on the Simoa® HD-X Analyzer software using a weighting factor of 1/y^2^ and a 4-parameter logistic curve fitting algorithm for P-Tau181. The N4PE test used eight-point calibration curves with 1/y^2^ weighting; a 4-parameter logistic fitting algorithm was used for NfL and GFAP, while a 5-parameter logistic fitting algorithm was used for Aβ 1-40 and Aβ 1-42. Quality control was performed with two levels after each calibration. The quality control material revealed variation between assays (expressed as % coefficient of variation) as follows: Aβ 1-40, 5% and 3% at approximate concentrations of 16 and 117 pg/mL; Aβ 1-42, 4% and 7% at approximate concentrations of 5.5 and 31 pg/mL; GFAP, 7% and 7% at approximate concentrations of 181 and 3702 pg/mL; NfL, 12% and 14% at approximate concentrations of 21 and 432 pg/mL; P-Tau181, 6% and 5% at approximate concentrations of 3.7 and 119 pg/mL. Selected plasma biomarkers were based on the assay available in this cohort at the time of this study.

### Gait Assessment

**Fig. 1:**
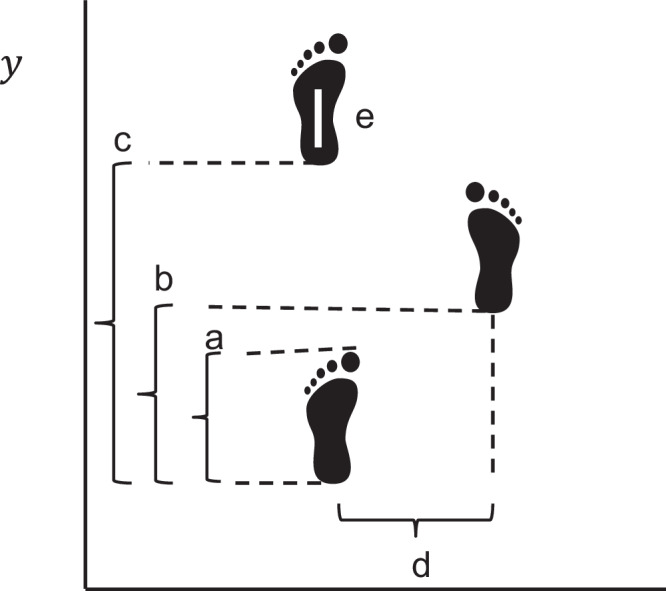
Visual representation of gait features. On a pressure sensitive mat, variables are derived through the pressure imprint of each foot and its x and y coordinates (location on the mat). In this figure **a**: maximum foot length, **b**: step length, **c**: stride length, **d**: step/stride width, **e**: stance center of pressure distance is the distance between the first and last contact of the foot as it lands on the ground.

Gait data was collected using a 10-meter long GAITrite^TM^ or Zeno^TM^ pressure sensitive walkway (ProtoKinetics). Participants were instructed to walk down the pressure sensitive walkway at a self-chosen pace. The pressure data obtained from the footfalls was processed using the Proto Kinetics Movement Analysis Software (PKMAS). Figure 1 shows a graphic representation of some of the spatial-temporal gait parameters used. Biologically meaningful variables capturing functionally relevant components of gait such as rhythm, pace, variability, and postural control were selected^28^. In brief, spatial features were captured using stride length. Temporal features were captured using absolute or percentage time of components of the gait cycle such as single support (time or percentage time spent on one foot per gait cycle). Spatial-temporal features were primarily summarized in velocity. Stability was also assessed using surrogate measures such as gait variability, captured using standard deviation (SD) of parameters (higher SD means greater variability)^40^ or center of pressure (COP) distance in stance or single support phases (shorter distance signifies abnormal foot placement). COP distance can be expressed as an absolute value or as a percentage (distance between the first and last contact of the foot expressed as a percentage of the foot length). Shorter single support time (time spent on one foot) also serves as a proxy for postural control. Those with poor balance tend to spend more time with both feet on the ground and less time with only one foot on the ground.

### Statistical analysis

There were 2654 individuals identified with gait variables, relevant covariates, and at least one plasma biomarker. We examined the data for outliers. After close review, 13 people were removed from the analysis since the data appeared to have been affected by a technical error during the gait recording session. The final sample size was 2641. Given the omitted cases had invalid data, were a small minority, and we retain a very large sample, we do not believe this introduced any bias. A cross-sectional analysis was performed using linear regression models utilizing the plasma biomarkers as predictors and the gait parameters as outcomes. From these models, the beta coefficient estimates, confidence intervals (CI), adjusted r-squared values, and *p*-values are presented. All models included age, sex, BMI, Charlson Comorbidity Index, and cognitive diagnosis as covariates. The plasma biomarkers were standardized into z-scores (i.e., subtract the mean and divide by the SD) to be able to compare plasma marker coefficients for each gait outcome. Age was centered (i.e., subtract mean) and divided by 10 to be able to interpret coefficients as changes by decade. Log transformations (base *e*) were applied to stride velocity SD, stride length SD, stride width SD, and stance time to improve linear model fit. The linear models were also run, separately, including plasma biomarker interactions with age, sex, CKD, and cognitive diagnosis. These models employed a complete cases approach to missing data in that they used only observations where all variables included in the given model were non-missing. Medical comorbidities were extracted using an algorithm that searches the medical record for established diagnoses to assess presence of CKD. The usual alpha level of 0.05 was utilized to determine statistical significance. All data preparation and analysis were performed using SAS version 9.4 (SAS Institute, Cary, NC) and R version 4.2.2 (R Foundation for Statistical Computing, Vienna, Austria).

### Reporting summary

Further information on research design is available in the Nature Portfolio Reporting Summary linked to this article.

## Results

This study included 2641 participants in the MCSA with available gait and plasma biomarker data. Cohort characteristics are summarized by cognitive diagnosis category in Supplementary Data 1. The age range of those included was 50 to 98 with a mean of 73 and a standard deviation [SD] of (10.4) years. There were slightly more males (53.3%) than females and 10.2% had CKD. Cognitive diagnosis was 78.4% cognitively unimpaired (CU) and 21.6% cognitively impaired (CI - mild cognitive impairment [MCI]: 20.7%, Dementia: 0.9%). The mean body mass index (BMI) was 28.4 (5.2) kg/m^2^ and the mean Charlson Comorbidity Index was 3.4 (3.3). Over three-quarters of the cohort was CU, and as expected, individuals with cognitive impairment were older, and had worse gait parameters and more abnormal plasma biomarker levels. Rates of menopause were high (98.3% in CU and 100% in CI) and rates of hormone replacement therapy use were overall low (approximately 10% in CU and under 5% in the CI groups), see Supplementary Table 1 for details.

**Fig. 2:**
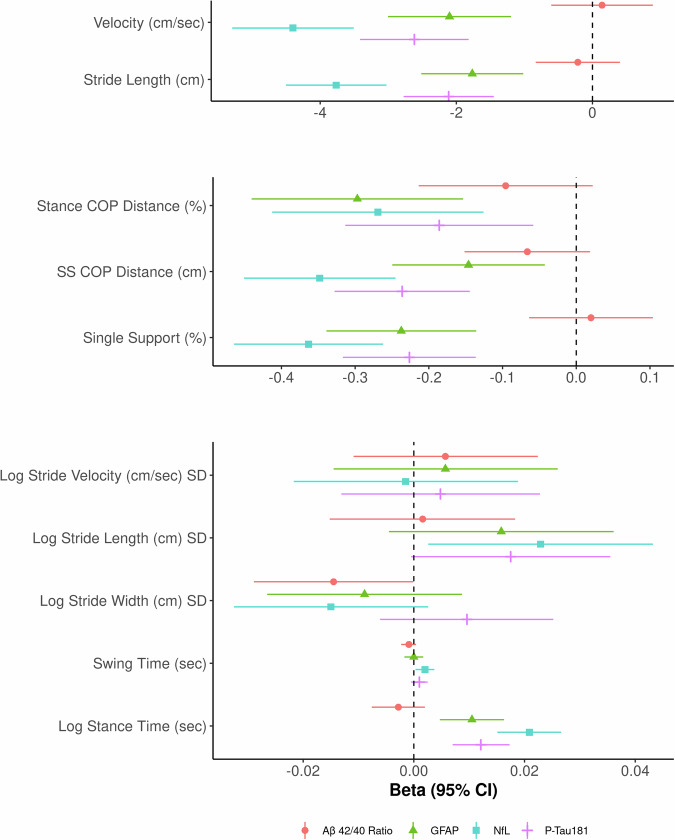
Forest Plots of Linear Regression Model Beta Coefficients and 95% Confidence Intervals. Dots correspond with beta coefficients from linear regression models adjusting for age, sex, body mass index, Charlson Comorbidity Index, and cognitive diagnosis, and the ends of the lines represent the 95% lower and upper confidence limits, respectively, from these models. Although the overall sample size was 2641, sample sizes used in the models ranged from 2628 to 2632 depending on the availability of the plasma marker variable included in each model. Coefficients where the 95% confidence interval does not cross the vertical dotted line are statistically significant. See supplementary data 2 for complete model results including *p*-values. SD Standard deviation, GFAP Glial fibrillary acidic protein, NfL Neurofilament light chain, COP Center of pressure, SS Single support.

All linear regression models were controlled for age, sex, BMI, Charlson Comorbidity Index, and cognitive diagnosis to assess the relationship between plasma biomarkers and gait parameters independent of these factors (results are summarized in Supplementary Data 2). Only stride width SD was found to be statistically significantly related to Aβ 42/40 ratio. On the other hand, multiple statistically significant associations were found for GFAP, NfL, and P-Tau181 with gait parameters. The most notable relationships were with velocity and stride length. Each SD increase in GFAP, NfL, and P-Tau181 was associated with a reduction in velocity of 2.100 (95% CI: −3.004, −1.196; *p* = 5.4 × 10^−6^), 4.400 (−5.292, −3.507; *p* = 9.5 × 10^−22^), and 2.617 (−3.414, −1.819; *p* = 1.5 × 10^−10^) cm per second, respectively. Similarly, each SD increase in GFAP, NfL, and P-Tau181, was associated with a 1.766 (−2.514, -1.017; *p* = 4.0 × 10^−6^), 3.764 (−4.503, −3.025; *p* = 4.2 × 10^−23^), and 2.112 (−2.775, −1.450; *p* = 4.6 × 10^−10^) cm shorter stride length, respectively. Additionally, stance center of pressure (COP) distance, single support (SS) COP distance and single support (%) decreased, and stance time increased, all of which is consistent with worse gait performance, with higher plasma biomarker (NfL, GFAP, P-Tau 181) levels in a statistically significant manner. NfL tended to have the strongest associations. Velocity and stride length denote pace, while single support time and center of pressure measurements are a surrogate for balance or postural control. Results are presented graphically as a forest plot in Fig. 2. Meeker and Guo et al have demonstrated possible utility of normalizing CSF P-Tau 181 by Aβ 40 in predicting AD neuropathology^41,42^. This approach has not been applied to plasma biomarker measurements, however we explored this ratio with results included in supplementary data 1. The predictive power of this ratio was overall weaker than using P-Tau 181 in adjusted models, hence this was not included in further analyses.

As detailed above, statistically significant associations were discovered between plasma biomarkers and gait parameters while controlling for age. However, to assess how advancing age influences these relationships, we evaluated whether there were any significant interactions between plasma biomarkers and age using interaction terms in the linear models (Supplementary Data 3). Evidence for interactions with the plasma biomarkers in predicting gait variables was observed, for age with GFAP, NfL, and P-Tau181. This means that while there was a relationship between higher plasma biomarkers of neurodegeneration and AD with worsening gait parameters independent of age, this relationship was compounded with advancing age. As an example, those at the average age had slower velocity, on average, by 2.975 (−4.122, −1.828; p = 4.0 × 10^−7^) and 1.854 (−2.762, −0.946; *p* = 6.4 × 10^−5^) cm/sec for each one SD increase in NfL and P-Tau181, respectively. Each of these associations strengthened with increasing age: with each additional decade of age above the mean, each SD increase in NfL corresponded to an additional reduction in velocity of 1.726 (−2.602, -0.849; *p* = 1.2 × 10^−4^) cm/sec and each SD increase in P-Tau181 was associated with an additional decrease in velocity of 1.583 (−2.488, −0.677; *p* = 6.1 × 10^−4^) cm/sec. Similarly, those at the average age for this sample had no observed relationship between GFAP and velocity (−0.298; −1.447, 0.851; *p* = 0.611), but with each additional decade above the mean, each SD increase in GFAP was associated with slower velocity by an additional 2.207 (-3.082, -1.333; *p* = 8.0 × 10^−7^) cm/sec. This strengthening of the association with increasing age between plasma markers GFAP, NfL, and P-Tau181 and gait outcomes was also seen for stride length, SS COP distance, stance time, and single support (%). These interaction model results, along with models noted previously without interaction terms, demonstrate that the association between plasma biomarkers and gait parameters exists across the age spectrum and is strengthened by advancing age. No statistically significant interactions with age were seen for Aβ 42/40 ratio.

The effect of sex was evaluated using models including sex interaction terms with the plasma biomarkers to predict gait parameters (Supplementary Data 4). Some differences in the associations were seen for women when compared to men. We found higher GFAP, NfL, and P-Tau181 to be associated with lower single support (%) and longer stance time for women, with these associations either attenuated or no longer statistically significant for men. For example, amongst women, each one SD increase in GFAP, NfL, and P-Tau181 were, respectively, associated with lower single support by 0.383 (-0.512, -0.253; *p* = 7.8 × 10^−9^), 0.467 (−0.599, -0.334; *p* = 5.8 × 10^−12^), and 0.360 (-0.502, -0.219; *p* = 6.5 × 10^−7^) with positive sex interaction effects indicating less association for men (GFAP: 0.294 [0.131, 0.457; *p* = 4.1 × 10^−4^]; NfL: 0.197 [0.034, 0.359; *p* = 0.018]; P-Tau181: 0.208 [0.038, 0.377; *p* = 0.017]). On the other hand, increased levels in these three biomarkers were all associated with lower stance COP distance in men with these associations not being present for women.

We also evaluated whether there was an effect of CKD or cognitive diagnosis on the observed relationship between plasma biomarkers and gait parameters. Very few statistically significant interactions were noted for cognitive diagnosis and CKD. For example, a higher P-Tau181 level was associated with increased stride velocity SD, stride length SD, and stride width SD for those without CKD but not for those with CKD. This suggests that observed relationships between plasma biomarkers and gait parameters were not driven by CKD. Higher NfL was associated with longer stance time and lower single support (%) for those CU with these associations strengthening for those with cognitive impairment pointing to more imbalance experienced by those with cognitive impairment. There were only four other statistically significant interaction terms, suggesting that the observed relationships between plasma biomarkers and gait parameters were not driven by cognitive impairment status. These models were controlled for age, medical comorbidity burden, sex, and BMI. For complete interaction results, see Supplementary Data 5-6.

## Discussion

In this cross-sectional study, we evaluated the relationships between plasma biomarkers of Alzheimer’s neuropathology and neurodegeneration with gait performance in a sample from a large population-based cohort of aging individuals. As hypothesized, higher levels of plasma biomarkers (GFAP, NfL, and P-Tau181) were associated with worse gait parameters in the pace, rhythm, and postural stability domains. These plasma biomarkers of neurodegeneration and AD neuropathology, which are commonly related to cognition, also appear to be markers of gait and balance decline in an aging cohort. These relationships were statistically significant even after controlling for age, sex, BMI, medical comorbidity burden, and cognitive diagnosis. NfL appeared to be most strongly related to gait parameters, however, associations were also observed for GFAP and P-Tau 181, suggesting that both axonal neurodegeneration and early AD neuropathology may contribute to motor decline in aging individuals. Prior studies have shown that worse gait performance is associated with multiple neuropathologies^43–46^. We also found a differential effect of plasma biomarkers on some gait parameters with increasing age and for women compared to men. We did not identify any specific gait parameter associated with a single specific plasma biomarker. Most of the gait parameters were associated with at least one, but often multiple, plasma biomarkers. Significant associations existed more so with pace and rhythm followed by postural stability and variability domains.

NfL had the strongest relationships with the gait variables. NfL is a non-specific marker of neuro-axonal injury^20,47^. NfL has been seen to increase with age^48^, which we accounted for by including age in our models. It is also elevated in aging individuals with poor physical function^26^ as well as in a variety of neuropathological processes, such as Alzheimer’s disease, Amyotrophic Lateral sclerosis (ALS), and Parkinson’s Disease^49,50^. With a low false positive rate, it is an effective tool to assess neurodegeneration, according to a recent multicenter validation study^51^. Elevated NfL may signal evolving pathological changes in neuronal gait circuits. We also found strong associations with GFAP, an astrocytic cytoskeletal protein that signals neurodegeneration and correlates with disability^19^, and P-Tau181, which is a marker of early Alzheimer’s disease-related neuropathological change^52–55^. No strong correlations between amyloid beta and gait metrics were seen in this cohort. This finding may be due to the sensitivity of the test assay used or the lack of biological significance of amyloid alone in gait change, however, the latter cannot be determined from the current study and will need further investigation. Plasma biomarkers provide an accessible and cost-effective global estimate of neurodegeneration in the brain, however, specific brain regions are known to play a role in motor function. While detailed neuroimaging is beyond the scope of this article, prior studies have implicated prefrontal, supplementary motor, sensory-motor cortices, hippocampus, basal ganglia, and their connecting white matter tracts in mediating gait decline in older adults^56–59^. Altered structural and functional connectivity of brain regions mediating attention and sensory-motor function has been associated with gait performance^60–62^. Whereas older adults who demonstrate resistance to motor and cognitive decline, termed “superagers” tend to have higher brain volumes^63^.

The effect of age on the relationship between plasma biomarker levels and gait is an important consideration^64,65^. In an aging cohort, elevated levels of NfL in individuals over 60 years of age were shown to correlate with atrophy signifying neuro-axonal injury, which could manifest as gait changes^64^. Our findings agreed with this report, in that advancing age tended to strengthen the relationships between elevated plasma biomarkers and worse gait measures in older individuals, potentially signaling the accumulating burden of neuropathological changes in gait pathways. These relationships also existed when controlling for age as a covariate, meaning that there is also an age-independent association between plasma biomarkers and gait parameters. Plasma biomarker elevation (P-Tau181, NfL, amyloid beta) has been shown to be associated with faster cognitive decline in MCI and dementia^16,54,66^. Future longitudinal studies could further assess whether a similar association is present between plasma biomarkers and progressive gait and balance decline seen in neurodegenerative diseases with careful consideration of other systemic mediators of gait dysfunction in older adults such as medical comorbidities, sarcopenia etcetera.

Sex modified the relationship of plasma markers and some gait parameters. After adjusting for age, BMI, Charlson Comorbidity Index, and cognitive diagnosis, GFAP, NfL, and P-Tau181 were associated with lower single support (%) for women, with these associations being weaker for men. A lower single support percentage implies that the percentage of the gait cycle spent on one foot is lower, more time is spent with both feet on the ground, pointing to gait instability. Women tend to have slower gait speed and a higher dual-task cost of walking with age^67–69^. Amongst men, higher GFAP, NfL, and P-Tau181 were associated with lower stance COP distance. This association was not observed among women. While the exact reason for the observed differences is not clear, it may be because distinct gait parameters capture aspects of gait biomechanics that differ between men and women. Rowe et al have previously shown using detailed motion analysis that men and women use different walking strategies^70^. Prior studies have also shown the effect of sex on biological aging, for example, that women experience lower accelerated aging, but higher frailty indices compared to men in one cohort^71^. Survival varies between men versus women experiencing reduced gait speed^72^. Our results demonstrate the need to consider sex when interpreting gait parameters in an aging individual and when assessing the clinical utility of plasma biomarkers to assess the impact of neurodegeneration on gait function.

Some of the statistically significant interactions we saw with CKD showed stronger associations amongst those without CKD suggesting that our findings are not driven by CKD and are more likely to be indicative of underlying neurodegeneration. As we continue to learn about plasma markers, the influences of medical comorbidities on plasma biomarkers, especially renal function that may affect serum protein constituents, should be examined when interpreting any plasma biomarker. Prior reports have shown that plasma biomarker levels may be affected by renal function and should be considered covariates in analyses examining their association with clinical parameters^73–75^.

Another important consideration is cognitive impairment and its contribution to gait and balance function. A population-based study in Spain showed that gait variability under dual-task conditions among older adults was in part explained by declining cognition^76^. To assess whether cognitive impairment was driving the observed relationship between plasma biomarkers and gait parameters, we examined models controlled for cognitive state and included a cognitive diagnosis interaction term (cognitively unimpaired or cognitively impaired). Associations between plasma biomarkers and gait were statistically significant after controlling for cognitive diagnosis. Findings suggest that additional factors, other than cognitive state alone drive gait impairment in older individuals and require further investigation.

Strengths of this study include a large sample from a well-characterized population-based cohort with concurrent measurement of plasma biomarkers of Alzheimer’s pathology (amyloid and tau) and non-specific neurodegeneration (NfL and GFAP) as well as multiple gait parameters. We evaluated the relationship between these plasma biomarkers and gait performance, laying the groundwork for future research assessing the relative contributions of neurodegeneration and AD neuropathology to motor function in older adults. Our study has a few important limitations. We did not include phosphorylated tau 217 (P-Tau217) in our analysis as it was not available in the assay we used. P-Tau217 measured in plasma may be a better marker of Alzheimer’s disease (AD) neuropathology^77^. Another limitation is that the plasma markers utilized here were measured using an immunoassay. Mass spectrometry assays may outperform other assays in terms of detecting abnormal amyloid levels and progression to AD^78^. This could at least partially explain the limited number of associations between Aβ 42/40 ratio and gait measures seen in this study. Future investigations will incorporate plasma biomarkers measured via mass spectrometry and association with other markers of neurodegeneration as well as vascular pathology. The population of Olmsted County is predominantly non-Hispanic white, which may limit the overall generalizability of the findings to more diverse groups. Future work should evaluate these findings in a diverse cohort.

In this cross-sectional assessment of a sample from a population-based cohort, multiple gait variables were found to be statistically significantly associated with plasma biomarkers of Alzheimer’s pathology and neurodegeneration with worse plasma marker levels being related to worse gait performance after controlling for age, sex, BMI, medical comorbidities and cognitive diagnosis. These associations were present for GFAP, NfL, and P-Tau181. Furthermore, some of these observed relationships were modified by sex and made stronger with advancing age.

## Supplementary information

Supplementary Material

Description of Additional Supplementary Files

Supplementary Data 1

Supplementary Data 2

Supplementary Data 3

Supplementary Data 4

Supplementary Data 5

Supplementary Data 6

Supplementary Data 7

Reporting Summary

## Data Availability

The data used in this manuscript is from the Mayo Clinic Study of Aging (MCSA). Due to the sensitive nature of this data, it is not publicly available. Anonymized data will be available upon request in accordance with Mayo Clinic and MCSA data-sharing protocols. For data sharing, you may refer to the following website for information: https://www.mayo.edu/research/centers-programs/alzheimers-disease-research-center/research-activities/mayo-clinic-study-aging/for-researchers/data-sharing-resources. The source data for Fig. 2 is in “Supplementary Data 7”.
