# Supplementary material for: Association of plasma biomarkers of Alzheimer’s pathology and neurodegeneration with gait performance in older adults
Source: Commun Med (Lond). 2025 Jan 16;5:19. doi: 10.1038/s43856-024-00713-6 (PMC11739691; doi:10.1038/s43856-024-00713-6)
Supplement: Supplementary file 1 — Supplementary Material [file 43856_2024_713_MOESM1_ESM.pdf]

Supplementary Files

Supplementary Table 1: Rates of menopause and hormone replacement therapy use

	CU (N=985)	CI (N=248)	Total (N=1233)	p value
**Menopause**				
N-Miss	102	18	120	0.051[1]
No	15 (1.7%)	0 (0.0%)	15 (1.3%)	
Yes	868 (98.3%)	230 (100.0%)	1098 (98.7%)	
**Estrogen Hormone Replacement Therapy**				
No	891 (90.5%)	240 (96.8%)	1131 (91.7%)	0.001[2]
Yes	94 (9.5%)	8 (3.2%)	102 (8.3%)	
**Progesterone Hormone Replacement Therapy**				
No	966 (98.1%)	248 (100.0%)	1214 (98.5%)	0.020[1]
Yes	19 (1.9%)	0 (0.0%)	19 (1.5%)	

CU: cognitively unimpaired, CI: cognitively impaired.
[1]Fisher's Exact Test for Count Data
[2]Pearson's Chi-squared test
