# Supplementary material for: Association of plasma biomarkers of Alzheimer’s pathology and neurodegeneration with gait performance in older adults
Source: Commun Med (Lond). 2025 Jan 16;5:19. doi: 10.1038/s43856-024-00713-6 (PMC11739691; doi:10.1038/s43856-024-00713-6)
Supplement: Supplementary file 2 — Description of Additional Supplementary Files [file 43856_2024_713_MOESM2_ESM.pdf]

Description of Additional Supplementary Files

File name- Supplementary Data 1

File description- Cohort characteristics are summarized by cognitive diagnosis category in Supplementary Data 1

File name- Supplementary Data 2

File description- Linear Regression Models Predicting Gait Parameters using Plasma Biomarkers

File name- Supplementary Data 3

File description-: Linear Regression Models Predicting Gait Parameters using Plasma Biomarkers with Age Interaction

File name- Supplementary Data 4

File description- Linear Regression Models Predicting Gait Parameters using Plasma Biomarkers with Sex Interaction

File name- Supplementary Data 5

File description- These models were controlled for age, medical 287 comorbidity burden, sex and BMI. For complete interaction results, see Supplementary Data 5- 6.

File name- Supplementary Data 6

File description- These models were controlled for age, medical 287 comorbidity burden, sex and BMI. For complete interaction results, see Supplementary Data 5- 6.

File name- Supplementary Data 7

File description- The source data for Figure 2 is in “Supplementary Data 7”.
