# Supplementary material for: Association of plasma biomarkers of Alzheimer’s pathology and neurodegeneration with gait performance in older adults
Source: Commun Med (Lond). 2025 Jan 16;5:19. doi: 10.1038/s43856-024-00713-6 (PMC11739691; doi:10.1038/s43856-024-00713-6)
Supplement: Supplementary file 10 — Reporting Summary [file 43856_2024_713_MOESM10_ESM.pdf]

nature portfolio

Corresponding author(s): Farwa Ali

Last updated by author(s): Nov 3, 2024

Reporting Summary

Nature Portfolio wishes to improve the reproducibility of the work that we publish. This form provides structure for consistency and transparency in reporting. For further information on Nature Portfolio policies, see our Editorial Policies and the Editorial Policy Checklist.

Statistics

For all statistical analyses, confirm that the following items are present in the figure legend, table legend, main text, or Methods section.

n/a	Confirmed	
☐	☒	The exact sample size (*n*) for each experimental group/condition, given as a discrete number and unit of measurement
☒	☐	A statement on whether measurements were taken from distinct samples or whether the same sample was measured repeatedly
☐	☒	The statistical test(s) used AND whether they are one- or two-sided<br>*Only common tests should be described solely by name; describe more complex techniques in the Methods section.*
☐	☒	A description of all covariates tested
☐	☒	A description of any assumptions or corrections, such as tests of normality and adjustment for multiple comparisons
☐	☒	A full description of the statistical parameters including central tendency (e.g. means) or other basic estimates (e.g. regression coefficient) AND variation (e.g. standard deviation) or associated estimates of uncertainty (e.g. confidence intervals)
☐	☒	For null hypothesis testing, the test statistic (e.g. *F*, *t*, *r*) with confidence intervals, effect sizes, degrees of freedom and *P* value noted<br>*Give P values as exact values whenever suitable.*
☒	☐	For Bayesian analysis, information on the choice of priors and Markov chain Monte Carlo settings
☒	☐	For hierarchical and complex designs, identification of the appropriate level for tests and full reporting of outcomes
☒	☐	Estimates of effect sizes (e.g. Cohen's *d*, Pearson's *r*), indicating how they were calculated

Our web collection on statistics for biologists contains articles on many of the points above.

Software and code

Policy information about availability of computer code

Data collection	Microsoft excel
Data analysis	SAS version 9.4 (SAS Institute, Cary, NC) and R version 4.2.2 (R Foundation for Statistical Computing, Vienna, Austria).

For manuscripts utilizing custom algorithms or software that are central to the research but not yet described in published literature, software must be made available to editors and reviewers. We strongly encourage code deposition in a community repository (e.g. GitHub). See the Nature Portfolio guidelines for submitting code & software for further information.

Data

Policy information about availability of data

All manuscripts must include a data availability statement. This statement should provide the following information, where applicable:
- Accession codes, unique identifiers, or web links for publicly available datasets
- A description of any restrictions on data availability
- For clinical datasets or third party data, please ensure that the statement adheres to our policy

Data will be available upon reasonable request.

Human research participants

Policy information about studies involving human research participants and Sex and Gender in Research.

Reporting on sex and gender	Sex was used as a covariate to assess its effect as a biologically significant variable. Interaction models using sex were also analyzed.
Population characteristics	Population based aging cohort.
Recruitment	All members of Olmsted county in Minnesota are invited. This limits selection bias since it is a population based random sampling, however it is conceivable that certain people are more likely to participate in such studies, these individuals may be healthier and more able to participate in longitudinal studies.
Ethics oversight	The study was approved by the Mayo Clinic IRB and all participants provided informed consent.

Note that full information on the approval of the study protocol must also be provided in the manuscript.

Field-specific reporting

Please select the one below that is the best fit for your research. If you are not sure, read the appropriate sections before making your selection.

☒ Life sciences ☐ Behavioural & social sciences ☐ Ecological, evolutionary & environmental sciences

For a reference copy of the document with all sections, see nature.com/documents/nr-reporting-summary-flat.pdf

Life sciences study design

All studies must disclose on these points even when the disclosure is negative.

Sample size	All participants in the Mayo Clinic Study of aging with gait parameters available were included.
Data exclusions	No data was excluded.
Replication	Findings were not replicated in this manuscript, however the methods are described in sufficient detail to allow replication by other groups or our group in a different cohort (future plan).
Randomization	There was no intervention delivered hence no randomization was performed.
Blinding	There was no intervention delivered hence no blinding was performed.

Reporting for specific materials, systems and methods

We require information from authors about some types of materials, experimental systems and methods used in many studies. Here, indicate whether each material, system or method listed is relevant to your study. If you are not sure if a list item applies to your research, read the appropriate section before selecting a response.

Materials & experimental systems

n/a	Involved in the study	
☒	☐	Antibodies
☒	☐	Eukaryotic cell lines
☒	☐	Palaeontology and archaeology
☒	☐	Animals and other organisms
☐	☒	Clinical data
☒	☐	Dual use research of concern

Methods

n/a	Involved in the study	
☒	☐	ChIP-seq
☒	☐	Flow cytometry
☒	☐	MRI-based neuroimaging

Clinical data

Policy information about clinical studies

All manuscripts should comply with the ICMJE guidelines for publication of clinical research and a completed CONSORT checklist must be included with all submissions.

Clinical trial registration	IRB registration number 14-004401

Study protocol	Described as methods in the manuscript.
Data collection	Participants were recruited from Olmsted country Minnesota in a population-based random selection manner. Participants have been recruited into this longitudinal cohort study on an ongoing basis for the past 10 years.
Outcomes	Plasma biomarkers of AD pathology and neurodegeneration were used to predict gait parameters.
